# Characterization of Sugarcane Mosaic Virus *Scmv1* and *Scmv2* Resistance Regions by Regional Association Analysis in Maize

**DOI:** 10.1371/journal.pone.0140617

**Published:** 2015-10-21

**Authors:** Pengfei Leng, Qing Ji, Yongfu Tao, Rania Ibrahim, Guangtang Pan, Mingliang Xu, Thomas Lübberstedt

**Affiliations:** 1 National Maize Improvement Center, China Agricultural University, Beijing, 100094, China; 2 Department of Agronomy, Iowa State University, Ames, Iowa, 50011, United States of America; 3 Maize Research Institute, Sichuan Agricultural University, Chengdu, Sichuan, 611130, China; Wuhan Botanical Garden of Chinese Academy of Sciences, CHINA

## Abstract

Sugarcane Mosaic Virus (SCMV) causes one of the most severe virus diseases in maize worldwide, resulting in reduced grain and forage yield in susceptible cultivars. In this study, two association panels consisting of 94 inbred lines each, from China and the U.S., were characterized for resistance to two isolates: SCMV-Seehausen and SCMV-BJ. The population structure of both association panels was analyzed using 3072 single nucleotide polymorphism (SNP) markers. The Chinese and the U.S. panel were both subdivided into two sub-populations, the latter comprised of Stiff Stalk Synthetic (SS) lines and Non Stiff Stalk Synthetic (NSS). The relative kinships were calculated using informative 2947 SNPs with minor allele frequency ≥ 5% and missing data ≤ 20% for the Chinese panel and 2841 SNPs with the same characteristics were used for the U.S. panel. The *Scmv1* region was genotyped using 7 single sequence repeat (SSR) and sequence-tagged site (STS) markers, and 12 SSR markers were used for the *Scmv2* region in the U.S. panel, while 5 of them were used for the Chinese panel. For all traits, a MLM (Mix Linear Model) controlling both population structure and relative kinship (*Q* + *K*) was used for association analysis. Three markers Trx-1, STS-11, and STS-12 located in the *Scmv1* region were strongly associated (*P* = 0.001) with SCMV resistance, and explained more than 16.0%, 10.6%, and 19.7% of phenotypic variation, respectively. 207FG003 located in the *Scmv2* region was significantly associated (*P* = 0.001) with SCMV resistance, and explained around 18.5% of phenotypic variation.

## Introduction

Sugarcane mosaic virus (SCMV), a member of potyviridae, causes chlorosis, stunting, and ultimately resulting in substantially reduced grain and forage yield in susceptible crops including sugarcane, maize, and sorghum [1–5]. Early infected plants may be totally barren. In China, SCMV was first reported in 1968, and is made responsible for a yield reduction of about 2500 kg per hectare in Henan province [6]. It is not possible to control SCMV by chemicals due to the non-persistent mode of virus transmission by aphids. Therefore, the most effective way to control SCMV infection is to cultivate resistant varieties, which contributes to sustainable crop production.

SCMV resistant lines have been identified, indicating that genetic resistance is indeed an economic way to control SCMV [7, 8]. Three lines (D21, D32, and FAP1360A), identified among 122 early maturing European maize inbred lines, displayed complete resistance to SCMV [7]. In the U.S., Pa405, B68, Oh7B, Mp339, GA209, and A239 were shown to be resistant to SCMV [9, 10], while Huangzaosi, Siyi, X178, and Hai9-21 displayed complete resistance to SCMV in China [11, 12]. All these lines were widely used for genetic analyses worldwide [5, 9].

SCMV resistance genes were first located in inbred line GA209 on both arms of chromosome 6 by use of translocation lines [13]. Research of maize resistance to SCMV was greatly facilitated with the development of molecular markers, such as Restriction Fragment Length Polymorphism (RFLP), Amplified Fragment Length Polymorphism (AFLP), and Single Sequence Repeat (SSR) markers [14–20]. The estimated number of SCMV resistance genes differs across populations, ranging from one to five. The first resistance gene *Mdm1* against maize dwarf mosaic virus (MDMV), which is closely linked to SCMV, was located near the centromere of chromosome 6 of Pa405 by flanking RFLP markers Umc85 and Bnl6.29 [21]. Using the cross between resistant line D32 and susceptible line D145, two major dominant resistance Quantitative Trait Loci (QTL) on chromosomes 3 and 6, and three minor QTL on chromosomes 1, 5, and 10, were identified [14, 16]. High-resolution mapping using progeny from the cross between FAP1360A (resistant) and F7 (susceptible) confirmed that *Scmv1* and *Scmv2* are two major SCMV resistance loci. Although both are required for complete resistance against SCMV, *Scmv1* has a stronger effect than *Scmv2*. *Scmv1* is located on the short arm of chromosome 6, and *Scmv2* near the centromere of chromosome 3. *Scmv1* suppresses symptoms at all developmental stages, *Scmv2* functions at later stages of infection [16, 22, 23]. Presence of resistance alleles at both loci, *Scmv1* and *Scmv2*, is crucial for complete SCMV resistance [15, 22]. According to the study of Yuan et al., there might be closely linked resistance genes within the *Scmv1* genome region [24].

When comparing QTL mapping results, *Scmv1* was assigned to a genetic region of about 7.4 cM, flanked by the SSR markers bnlg161 and phi077 on chromosome 6, and *Scmv2* was placed in a 17 cM region, flanked by bnlg1456 and bnlg1035 on chromosome 3 [16, 18, 24]. Recently, *Scmv1* was fine mapped by a segregating population derived from near-isogenic lines, and assigned to a 59.21 kb interval [25]. Using a large isogenic mapping population segregating for the *Scmv2* region, *Scmv2* was fine mapped to a region of 0.28 cM, covering a physical distance of 1.3426 Mb, and four genes were suggested as positional candidates [26].

The two most commonly used methods to dissect complex traits in plants are linkage analysis and association mapping. Linkage analysis uses a well characterized pedigree to identify the non-random association between genotype and phenotype, whereas association mapping utilizes ancestral recombination in unrelated individuals and linkage disequilibrium (LD) to identify associations between genotype and phenotype [27]. Association analysis, based on LD, is a method that can be used to identify the actual genes represented by QTL based on the relationship of specific sequence polymorphisms in candidate genes and phenotypic variation [28]. Success of gene-based association studies depends on the candidate gene(s) chosen for a particular phenotypic trait. The first candidate gene-based association mapping study in plants, associating individual *Dwarf8* polymorphisms with maize flowering time [28], was followed by numerous studies in maize [27, 29–33] and in other crops [34, 35]. Association analyses succeeded to finding new genes and also contributed to identify valuable alleles. Gene-based association studies ultimately lead to identification of quantitative trait polymorphisms (QTPs) with causal genetic effects on agronomic or resistance traits, which can be converted into functional markers [4, 36].

The aims of this study were, to (1) identify candidate SCMV resistance genes in the *Scmv1* and *Scmv2* regions by candidate gene region based association analysis, using two maize association panels; and (2) to compare results across panels and virus isolates.

## Results

### Phenotypic data

As the phenotypic data of the Chinese panel had been published by Tao et al. (2013), they are not displayed in this manuscript. Significant variation for all SCMV-related traits was found among lines, experiments, and also the interaction for the U.S. panel. Estimates of the variances for genetics and the related interactions were highly significant (*P* = 0.01) for two SCMV isolates resistance on all scoring dates (Table 1, S1 Fig).

**Table 1:** Estimates of variance components for the U.S. panel inoculated with SCMV-Seehausen and SCMV-BJ.

Source	DF	Variance components	
		SCMV-Seehausen	SCMV-BJ
**Experiment**	1	0.0001	0.001*
**Line**	93	0.138**	0.068**
**Experiment × Line**	93	0.149**	0.020**

DF, degree of freedom

**, *P* = 0.01

*, *P* = 0.05.

The mean percentage of plants showing symptoms after inoculation with SCMV-BJ varied from 0.20 at 1 WPI to 0.54 at 4 WPI in Experiment 1, and from 0.23 to 0.65 in Experiment 2 (Table 2). The broad sense heritability (*H* ^2^) ranged from 0.69 to 0.75 from 1 to 4 WPI for the SCMV-BJ isolate. The *H* ^2^ estimates for SCMV-Seehausen ranged from 0.80 to 0.92, which indicates that the U.S. panel is suitable for association analysis of SCMV resistance. The mean percentage of plants with symptoms across time points varied from 0.10 to 0.37 and 0.17 to 0.4 in Experiments 1 and 2, respectively.

**Table 2:** Descriptive statistics and broad sense heritability for the U.S. panel inoculated with SCMV-Seehausen and SCMV-BJ.

Category	Mean ± SE			*H* ^2^ ± SE
	Experiment-1	Experiment-2	Across Experiments	
**SCMV-BJ**				
**1 WPI**	0.20±0.02	0.23±0.03	0.22±0.02	0.69±0.04
**2 WPI**	0.35±0.03	0.50±0.04	0.42±0.02	0.67±0.05
**3 WPI**	0.49±0.04	0.59±0.04	0.54±0.03	0.75±0.04
**4 WPI**	0.54±0.04	0.65±0.03	0.60±0.03	0.75±0.04
**SCMV-Seehausen**				
**1 WPI**	0.10±0.02	0.17±0.03	0.14±0.02	0.80±0.04
**2 WPI**	0.18±0.03	0.32±0.03	0.25±0.03	0.83±0.03
**3 WPI**	0.30±0.03	0.45±0.03	0.37±0.03	0.90±0.02
**4 WPI**	0.37±0.04	0.47±0.04	0.42±0.03	0.92±0.02

WPI: week post inoculation

*H* ^2^: broad sense heritability.

### SNP performance and quality

All SNPs were well distributed across the 10 maize chromosomes, and coverage ranged from 188 SNPs on chromosome 10 to 448 SNPs on chromosome 4 (Table 3). Of the 3072 maize SNPs, 3053 (99.38%) in the Chinese panel and 3042 (99.02%) in the U.S. panel were successfully called in 94 lines with missing data below 20%. Only 3.61% (111/3072) of the SNPs used for the Chinese panel and 6.54% (201/3072) of the SNPs used for the U.S. panel had a minor allele frequencies (MAF) below 0.05. The MAFs of 3072 SNPs in the Chinese panel averaged 0.35, and about 72% of the SNPs had an MAF greater than 0.30. In the U.S. panel, the MAFs averaged 0.35 and about 70% of the SNPs had a MAF greater than 0.30. Gene diversity ranged from 0.1 to 0.5, with an average of 0.43 for the Chinese panel and 0.42 for the U.S. panel. Polymorphism information content (PIC) values ranged from 0.095 to 0.38, with an average of 0.34 and 0.33, respectively. A final total of 2947 and 2841 SNPs were used for the Chinese and the U.S. panel for subsequent data analysis, respectively.

**Table 3:** Summary of 3072 SNPs used in this study.

Chromosome	SNP Number	Minor Allele Frequency (≥0.05)	
		Chinese panel	U.S. panel
**1**	384	371	352
**2**	308	293	275
**3**	231	223	219
**4**	448	429	422
**5**	319	303	297
**6**	281	275	267
**7**	266	259	253
**8**	434	426	409
**9**	213	204	202
**10**	188	178	175
**Total**	3072	2961	2871

### Population Structure

**Fig 1:**
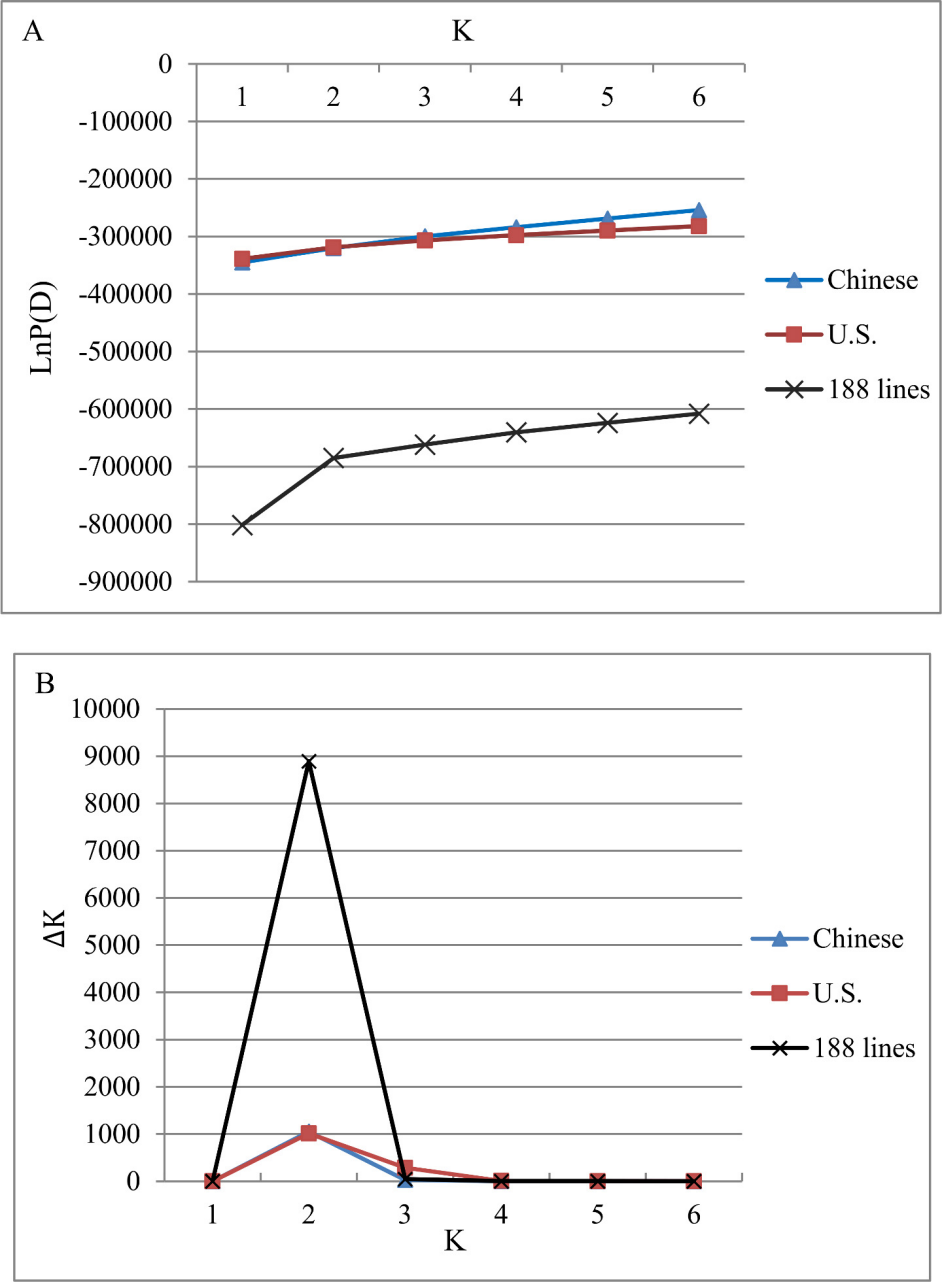
Population structure analysis of the Chinese and U.S. association panels and all the 188 lines. Plots of (A) LnP (D), and (B) ΔK based on 3072 SNPs.

According to the STRUCTURE results, the likelihood values of LnP(D) (Estimated Ln(probability of the data)) for both panels increased continuously and the most significant change was observed when K increased from 1 to 2 (Fig 1A). Using the ad hoc ΔK method [37], a distinct peak at ΔK = 2 was obtained (Fig 1B). Both findings suggest that the best number of sub-groups in both panels is 2. 64 lines of the Chinese panel were assigned to one group consisting of REID, LANCASTER, and the Zi330 subgroup (S1 Table). The remaining 30 lines were assigned to another group containing the Tangsipingtou and P subgroup, and 20 “mixed” lines had membership probabilities lower than 0.75 to the other heterotic groups. For the U.S. panel, the first group included 20 lines, all of which are SS lines. The second group contained 74 lines, most of which are NSS lines, tropical, sub-tropical lines, and mixed lines (S1 Table).

A joint population structure analysis across all 188 lines was conducted, and the most significant change was found at K = 2 (Fig 1A), as well as a sharp peak at ΔK = 2 (Fig 1B). Both parameters suggest that the best number of sub-groups for all 188 lines is two, in this case dividing all lines into the 94 Chinese and 94 U.S. lines. Furthermore, the PCA (principal components analysis) obtained from the Genomic Association and Prediction Integrated Tool (GAPIT) indicated a closer relationship of inbreds within rather than between the Chinese and the U.S. panels (S2 Fig). PC1 and PC2 explained 6.5% and 4.9% of the total SNP variance, respectively.

### Relative kinship

**Fig 2:**
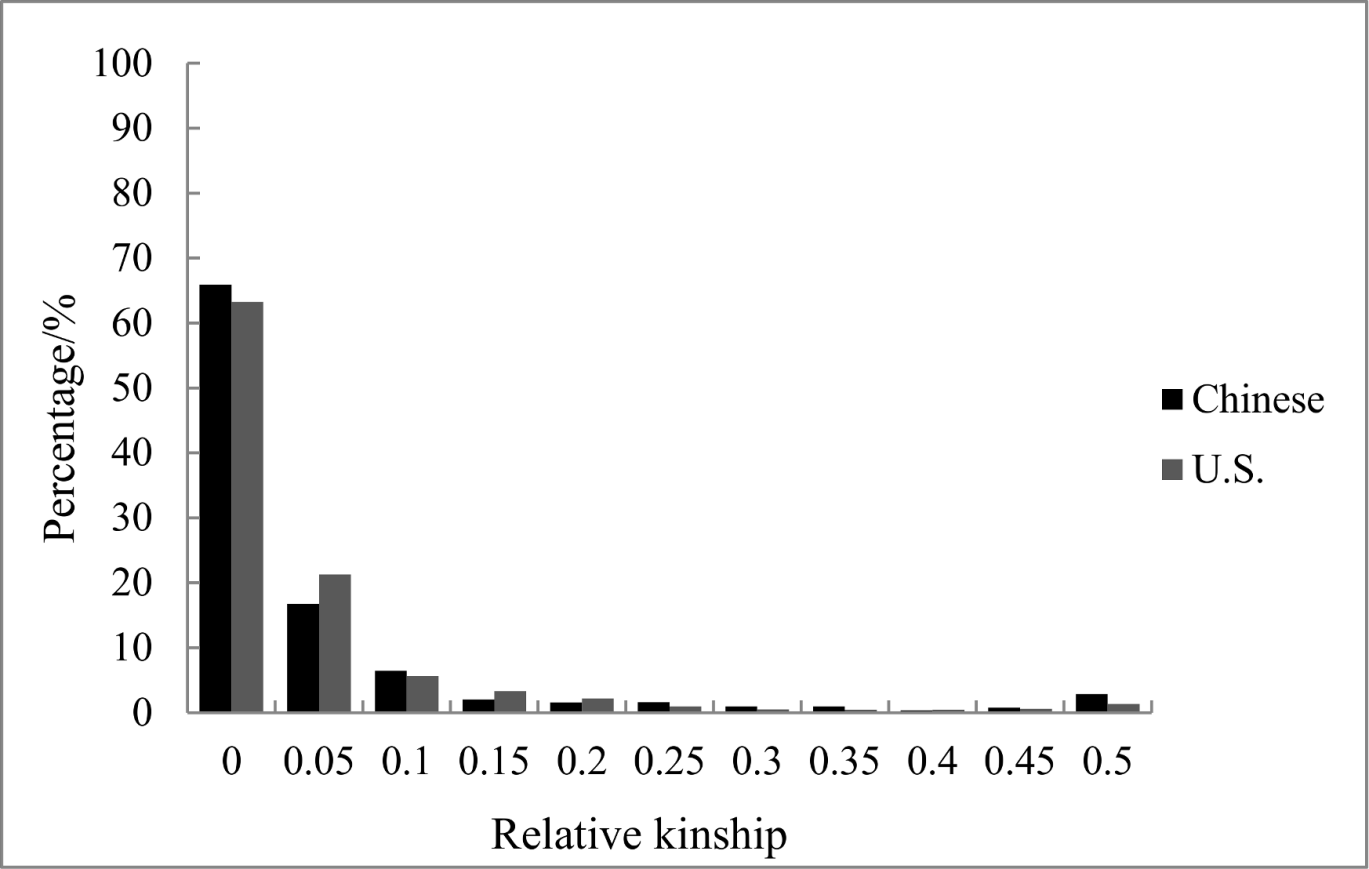
Distribution of pairwise relative kinship estimated between 2 × 94 inbred lines. Kinship estimates are based on 2947 (Chinese) and 2841 (U.S.) SNPs. For simplicity, only percentages of relative kinship ranging from 0 to 0.50 are shown.

In this study, 2947 informative SNP markers with MAF>0.05 and missing data below 20% were used to estimate the relative kinship of 94 Chinese lines and 2841 SNPs were used for the U.S. panel. Relative kinship estimates based on the SNP data showed that more than 60% of the pairwise kinship estimates in the two panels were equal to 0, and the remaining estimates ranged from 0.32% to 16.73% (Chinese panel) and 0.43% to 21.28% (U.S. panel), with a continuously decreasing number of pairs falling in higher estimate categories (Fig 2). The kinship analysis indicates that most lines within the two panels had no or very weak relationships with the other lines.

### Association mapping

#### SNPs associated with SCMV resistance

SNP marker PZE-110008811 located on chromosome 10 (6537076) was found to be associated with SCMV-BJ resistance in Experiment 2 (*P*<0.00002). SNP marker PZE-106020499 (Chr. 6: 16167793) was about 1.92 Mb away and the closest linked SNP to the *Scmv1* region in our study, with *P* values ranging from 0.25 to 0.78. PZE-103081176 (Chr. 3: 134867390) was the closest linked SNP to the *Scmv2* region (0.95 Mb away), with a *P* value ranging from 0.00368 to 0.82.

#### *Scmv1* region

**Fig 3:**
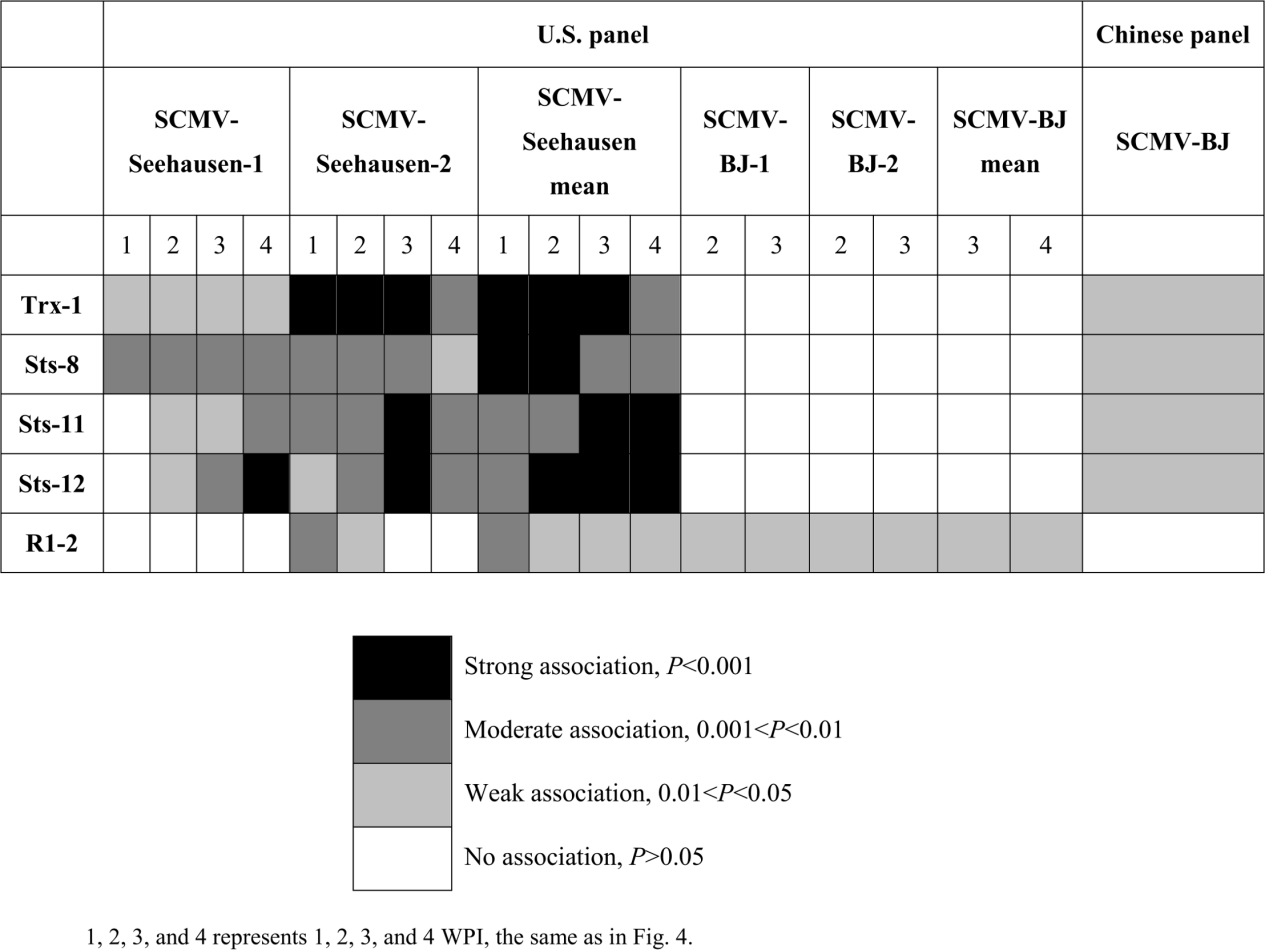
Markers significantly associated with SCMV resistance in the *Scmv1* region. 1, 2, 3, and 4 represents 1, 2, 3, and 4 WPI, the same as in Fig 4.

Three STS markers, STS-8 STS-11, STS-12, and Trx-1 in the *Scmv1* region were significantly associated with SCMV resistance for isolate SCMV-Seehausen (*P* = 0.001) in the U.S. panel (Fig 3). Both were also associated with SCMV-BJ resistance in the Chinese panel. Trx-1 was associated with SCMV resistance at 1, 2, and 3 WPI in Experiment 2, and explained 16.0 to 28.3% of the phenotypic variation. STS-11 was strongly associated with SCMV resistance (SCMV-Seehausen) in Experiment 2 at 3 WPI, explaining 22.6% phenotypic variation. STS-12 was associated with SCMV resistance (SCMV-Seehausen) 4 WPI in Experiment 1 and 3 WPI in Experiment 2 (*P* = 0.001), explaining 19.7 to 31.2% phenotypic variation. STS-8, which was almost always moderately (*P* = 0.01) associated with SCMV resistance (SCMV-Seehausen) across all four weeks of scoring, and explained 19.2 to 26.7% of the phenotypic variation. STS-8, STS-11, STS-12 together with Trx-1 were significantly associated with SCMV resistance, when using mean trait values across both Experiments. All four markers mentioned above were associated with SCMV-BJ resistance in the Chinese panel (*P* = 0.05). No significant association was found for SCMV-BJ resistance in the U.S. panel.

When all 188 lines were combined, Trx-1 was found to be associated with SCMV-BJ resistance at 1 WPI in Experiment 2 (*P* = 0.05), while STS-12 was also associated across both experiments.

#### *Scmv2* region

**Fig 4:**
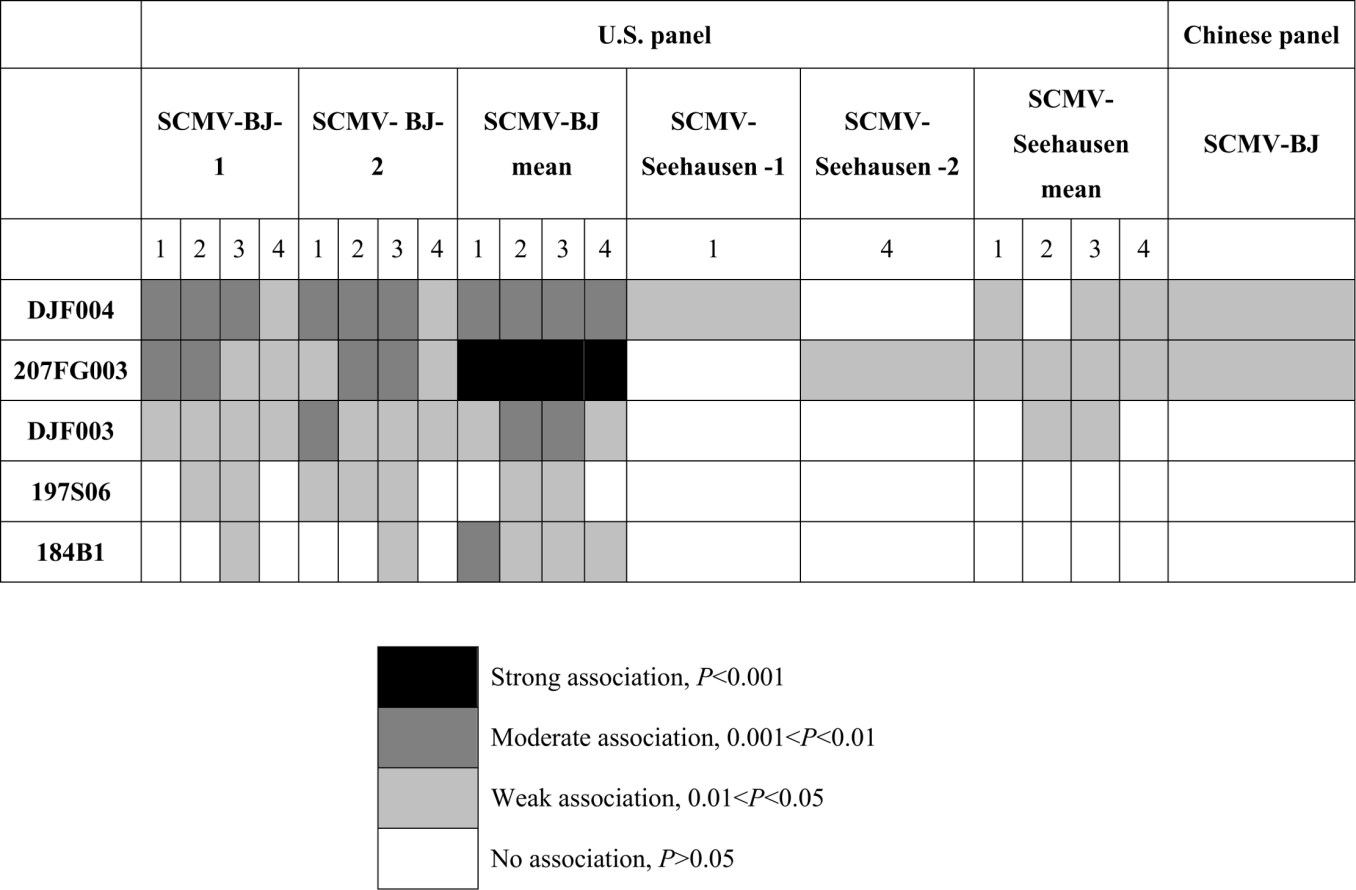
Markers significantly associated with SCMV resistance in the *Scmv2* region.

207FG003 was highly significantly associated (*P* = 0.001) with SCMV resistance (SCMV-BJ isolate) (Fig 4). 207FG003 was significantly associated with SCMV (SCMV-BJ isolate) at 1 and 2 WPI in Experiment 1 and 2, 3 WPI in Experiment 2, and explained 14.8% and 19.6% of the phenotypic variation. This SNP marker was also strongly associated with SCMV (SCMV-BJ isolate) when using mean trait values across both experiments. DJF004, developed for *Scmv2* fine mapping and co-segregating with *Scmv2*, was moderately associated with SCMV resistance (SCMV-BJ isolate) for the first three time points of scoring in both Experiments, and explained phenotypic variation from 21.7% to 26.2%. It was associated with SCMV-BJ resistance during all four weeks of testing. DJF003 was significantly (*P* = 0.05) associated with SCMV resistance (SCMV-BJ isolate) for the first week after inoculation, and explained 13.3% of the phenotypic variation. In addition, 197S06, was associated with SCMV-BJ resistance at 3 WPI (*P* = 0.05). Marker 184B1 was associated with SCMV resistance (SCMV-BJ isolate) (*P* = 0.05) when using mean trait values across both experiments. In the U.S. panel, DJF004 and 207FG003 were associated at 1 WPI and 4 WPI with SCMV resistance (SCMV-Seehausen) in both experiments, respectively, and explained 17.5% and 9.4% phenotypic variation.

In the Chinese panel, those markers associated with SCMV resistance in the U.S. panel such as DJF004 and 207FG003, were also associated with SCMV-BJ resistance (*P* = 0.05), and explained 15% and 12% phenotypic variation. DJF004 in the *Scmv2* region was associated with SCMV-BJ resistance at 1 WPI across both Experiments (*P* = 0.05), when combining the 188 lines.

## Discussion

### Genetic diversity and population structure

Genetic relatedness among individuals in an association panel is a key factor causing spurious associations. In this study, population structure of the two panels was established using 3072 SNP markers. Population structure (Q-matrix), estimated using STRUCTURE and expressed as membership probabilities, is one way to correct spurious associations due to genetic relatedness.

The Chinese 94 lines were divided to two sub-populations, consistent with groups known to have diverged in modern breeding history (flint and non-flint groups), the results were consistent with identified by Tao et al. [25], who used a finer differentiation. The same Chinese panel was divided into six subgroups based on 70 SSR markers: Lancaster (5), P (7), Reid (7), Zi330 (9), Tang SPT (12) and a mixed group (54) [25]. This may be due to the smaller number of markers and weak genetic differentiation within groups [38]. Hamblin assessed population structure by comparing 89 SSRs and 847 SNPs, showing that SSRs performed better at clustering individuals into populations than SNPs, and the population structure assessed was consistent [39]. It was suggested that over 10 times more SNPs than SSRs should be used to estimate relative kinship in maize association analysis [40]. The P1 group included four subgroups: Reid, Lancaster, Zi330, and some mixed lines, P2 was organized into two known heterotic groups: Tang SPT and P populations. These results agreed well with previous studies on Chinese maize inbred lines that separated Chinese lines into six [41] or four groups [42].

For the U.S. panel, K = 2 was the best possible partition in agreement with known pedigree history and geographic origin. The 94 lines could be assigned to P3 (26) and P4 (68) subgroups, most lines (>17) in the first group were known SS lines, and the P4 group contained more than 19 known NSS and 10 Tropical/Subtropical lines. 15 lines from the P4 group were collected from Germany, Canada, Spain, South Africa, Korea and Thailand, while the rest are public lines (including selected 18 PVP (Plant Variety Protection) lines and 17 NAM (Nested Association Mapping) lines). Most of the public lines in this study were described as important founder elite lines. B73, a known SS line, was found within the P3 subpopulation, whereas Mo17 (NSS) was member of the larger P4 subpopulation, and most CIMMYT maize lines (CMLs), like CML69, CML228, and CML333, were grouped into P4 in this study. Our results were consistent with other research on the U.S. inbred lines genetic structure and diversity inferred from DNA microsatellites [43].

### SCMV resistance assays

Heritability values ranged from 0.69 to 0.75 (SCMV-BJ) and 0.80 to 0.92 (SCMV-Seehausen) from 1 to 4 WPI. Previous studies have shown similar ranges of heritabilies for SCMV resistance at various stages of infection, ranging from 0.77 to 0.94 from the first to the final scoring date [16]. Resistance levels of lines in our panels were generally consistent with previous studies, such as susceptibility of F7, D145, B73, and Mo17 [6, 14, 15] and resistance of FAP1360A, Siyi, and Huangzao4 [6, 15]. Within the U.S. panel, most lines responded consistently to both SCMV isolates. Most CML inbreds showed a higher percentage of infected plants for SCMV-BJ than for SCMV-Seehausen, such as CML322 (14% / 0%), CML52 (100% / 38%) and CML247 (100% / 75%), respectively. Surprisingly, resistant lines 10940, D21, D32, and Pa405 [7, 9], were resistant to SCMV-Seehausen, but were not completely resistant to SCMV-BJ, 55%, 86%, 75% and 15% plants were susceptible for these lines respectively. Four dent inbreds D06, D09, FAP1396A, and R2306 were completely resistant to SCMV-Seehausen but susceptible to SCMV-BJ, while they were reported to be partially resistant to SCMV-Seehausen [7]. Thus, SCMV-BJ appears to be substantially more infectious than SCMV-Seehausen. Consistent with these findings, inbred 10940 was resistant to SCMV-Seehausen, but susceptible to an Italian isolate of MDMV in a previous study [44].

### Associations in the *Scmv1* region

*Scmv1* was reported to account for high levels of phenotypic variation (up to 56%) [16], and to be the key gene in SCMV resistance. *Scmv1* was repeatedly detected in different inbred lines [16, 18]. In this study, four markers, STS-8, STS-11, STS-12 and Trx-1, were strongly associated with SCMV-BJ and SCMV-Seehausen resistance in the *Scmv1* region, all of them were close to a *Thioredoxin h-type* gene. Significant associations between two SNPs and SCMV-BJ resistance supported that *Zmtrx-h* is the primary candidate for *Scmv1* [25]. Different from their study using the same panel of 94 Chinese inbreds, 3072 SNPs were used to estimate the population structure in our study and the resulting Q matrix was used for SCMV-BJ and SCMV-Seehausen association analysis. Two SCMV isolates were used in our *Scmv1* association analysis. In the U.S. association panel, Trx-1, STS-11, and STS-12 were strongly associated with resistance to SCMV isolate Seehausen, while no marker was significantly associated with resistance to SCMV-BJ isolate in this study. While our results were generally consistent with Tao et al. [25], confirming that *Zmtrx-h* is very likely the *Scmv1* gene. Flanking marker R1-2, located outside the fine mapped *Scmv1* region, was associated with resistance to SCMV-BJ and -Seehausen isolates in the U.S. panel.

In plants, *Trx-*h seems to be involved in multiple functions. The mechanism of thiol- redox control is emerging as a regulatory mechanism in several signal transduction pathways. Thioredoxin is a master regulator of cellular redox status [45], the role of thioredoxin in defense against hydrogen peroxide was elucidated by using a *Escherichia coli* thioredoxin-deficient mutant. H-type thioredoxin was reported to be involved in the resistance of tobacco to virus infection and abiotic oxidative stress. A *Trx-*h like gene predicted to encode an h-type *Trx* in tobacco (*Nicotiana tabacum*) enhanced tobacco resistance to Tobacco mosaic virus and Cucumber mosaic virus by its overexpression, and also conferred resistance to oxidative stress induced by paraquat [46]. However, it seems that STS-8, STS-11, STS-12, and Trx-1 associated with SCMV-Seehausen resistance while lack of association with SCMV-BJ resistance, this results might support the findings of Yuan et al. (2003) that presence of at least two closely linked resistance genes/QTL in the *Scmv1* region.

### Associations in the *Scmv2* region

*Scmv2* was fine mapped to a physical region spanning from the beginning of BAC clone c0483H04 (including DJF003) to the end of b0239F02 (including bnlg1601), covering a distance of 1.3426 Mb [26]. Only two well characterized genes, glutathione synthetase (*GS*) and auxin binding protein 1 (*ABP1*), were found among the unigenes, located at the overlap of BAC clones c0023O09/b0645C18 and b0645C18/c0281K07, respectively. The gene coding hypothetical protein SORBIDRAFT_08g016700 (GRMZM081350) was located at the overlap of c0078M13/c0023O09, and the Putative uncharacterized protein (GRMZM15599) and unknown (GRMZM160862) were located inside the BAC clone c0023O09. In this study, 207FG003 was designed according to the sequence of *ABP1*, and located at its 3’ terminus. The closest gene to DJF004 was *GS*, which was reported to play an essential role in many cellular processes as development, growth and usually early stress responses [47]. DJF004 co-segregated with *Scmv2*, and three recombinants between DJF003 and DJF004 and three between DJF004 and bnlg1601 were left on either side of *Scmv2* locus. DJF003 was another marker has weakly significant (*P* = 0.05) associated with SCMV-BJ and SCMV-Seehausen, closest linked to the abovementioned hypothetical protein. Again, 207FG003 was associated with SCMV-BJ resistance under two experiments with a very significant relationship (*P* = 0.01). In summary, 207FG003 was always significantly associated with SCMV-BJ at each time point, and DJF004 was found to be significantly associated with both SCMV-BJ and SCMV-Seehausen. The other marker 197S06 having significant associations with SCMV-BJ resistance at some time points, are closest linked to *ABP1*, an unknown and a hypothetical protein. Based on our findings that 207FG003 and DJF004 were significantly associated with SCMV resistance, both *ABP1* and *GS* have to be considered as candidates for *Scmv2*.

*ABP1* is an important receptor for auxin, a hormone involved in almost every aspect of plant growth. *ABP1* plays an important role in cell expansion [48], cell cycle, clathrin-dependent endocytosis [49], and cytoskeleton rearrangement [50]. *ABP1* is required for organized cell elongation and division in the embryogenesis in Arabidopsis and homozygous null mutants are embryonic lethal [51], and also related to auxin-mediated ion transport and osmoregulation at the plasma membrane [52]. As an ancient protein, *ABP1* is present from bacteria, algae to flowering plants, and obtained the endoplasmic reticulum retention motif only recently [53]. *GS* is essential for glutathione biosynthesis, and glutathione has been shown to be involved in the protection of plants against various types of stress [47, 54] as well as in the induction of defense-related genes [55]. Silencing of an EIL2 transcription factor gene and a *GS* gene was found to compromise the resistance of *Nicotiana megalosiphon* to *P*. *hyoscyami* f. sp. tabacina [56].

Cell-to-cell movement of potyviruses through plasmodesmata is required for virus spread [57]. Intracellular transport along the host membrane and the cytoskeletal network has been proposed to be carried out in form of 6K2-vesicles [58, 59]. As *Scmv2* activity occurs at late stages of SCMV infection [23], most likely it interferes with long distance movement. Cell cytoskeleton rearrangements induced by *ABP1* might change the structure of vascular bundles and block SCMV systemic transport [50, 60].

### Locus on Chromosome 10 associated with SCMV resistance

Using ca. 3000 SNP markers, a single SNP marker PZE-110008811 located on chromosome 10 was found to be associated with SCMV-BJ resistance (*P*<0.00002) in the U.S. panel. Xia et al. (1999) reported two major resistance QTL on chromosomes 3 and 6, and three minor QTL, including one on chromosomes 10. By using 184 F_2_ individuals derived from the cross of Huangzao4 (R) and Ye107 (S), a major QTL on chromosome 10 explaining 15.3% to 15.8% phenotypic variance was detected at four developmental stages (seedling, elongation, anthesis and grain-filling) [61]. However, the SNP marker PZE-110008811 on chromosome 10 (6537076) was not in the same region as previously reported to confer MDMV, WSMV, or SCMV resistance [62, 63]

## Conclusion

We identified significant markers associated with SCMV resistance in both the *Scmv1* and *Scmv2* regions within different association panels and using two different SCMV isolates. The closely linked markers will be useful for breeding new SCMV- and likely potyvirus-resistant lines, as *Scmv1* and *Scmv2* likely act pleiotropic [20].

## Materials and Methods

### Association panels

A collection of 94 Chinese maize inbred lines was established, representing considerable germplasm diversity available in Chinese breeding programs [25]. A total of 94 European and U.S. inbred lines (U.S. panel) were collected as 2^nd^ association panel (S2 Table), comprising current as well as historically important lines from both temperate and tropical programs, including popcorn and sweet corn lines with genetically distinct breeding histories, as well as known potyvirus resistant lines, such as FAP1360A, Pa405, D32, D21, B68, and Oh7B.

### Potyvirus resistance assays

The resistance of Chinese lines was tested against the SCMV-BJ isolate. Assays were performed twice in growth chamber experiments with 16 h of light (500 μE/s/m^2^) per day, relative humidity of 90%, 23°C for the light and 20°C for the dark period at China Agricultural University (Beijing, China). The U.S. panel was tested twice each against SCMV-Seehausen and the SCMV-BJ isolate in growth chamber experiments with 16 h of light (600 μE/s/m^2^) per day, 22°C for the light and 20°C for the dark period as Experiment 1 and another chamber with 16 h of light (600 μE/s/m^2^) per day, 25°C for the light and 20°C for the dark period as Experiment 2 at Iowa State University (Ames, IA, USA). Plants were sown in small pots, and two independent experiments were conducted, each experiment consisted of two replicates. FAP1360A and F7 were used as resistant and susceptible controls. Each line was represented by nine plants in each replicate. Experimental design was a randomized complete block design.

Inoculation was conducted at three-leaf stage. Fresh young leaf tissue with typical mosaic symptoms was harvested from SCMV infected susceptible F7 plants and ground in five times 20 mM phosphate buffer (pH 7.0) using pestle and mortar. Carborundum was added to the sap before inoculation. Plants were inoculated artificially by rubbing both sides of leaves with fresh prepared sap. Plants that did not show symptoms one week post first inoculation, were inoculated for a second time. Phenotypic data for the U.S. panel were collected weekly until 4 WPI, whereas the Chinese panel was only scored 1 WPI. Plants were classified as symptomatic (S) or not showing symptoms. For each inbred line disease incidence was measured as the percentage of infected plants. Most lines showed disease symptoms one week after inoculation in both the Chinese and the U.S. panel. Therefore, data from replications were combined to provide line means for association tests. For each of the two panels and the different traits including U.S. panel inoculated with SCMV-Seehausen, U.S. panel inoculated with SCMV-BJ, and Chinese panel inoculated with SCMV-BJ, the phenotypic percentage values of individual lines ranged from 0 to 100% for all time points.

### SNP genotyping and analysis

All DNAs were isolated using a standard CTAB extraction method with modifications [64]. Both Chinese and U.S. panels were genotyped by using GoldenGate assays (Illumina, San Diego, CA, USA) containing a set of 3072 SNP markers (S3 Table) evenly distributed throughout the maize genome. SNP genotyping was performed on an Illumina iScan (Illumina, San Diego, CA, USA) at the National Maize Improvement Center (China Agricultural University, Beijing, China) using the method supported by Illumina [65]. SNP data were analyzed using the Illumina GenomeStudio genotyping software (http://www.illumina.com/software/genomestudio_software.ilmn) which clusters and calls data automatically, allowing inspection of data prior to further analysis.

### Population structure analysis

The number of alleles, MAFs, missing data, heterozygosity, gene diversity and PIC were calculated using Powermarker 3.25 [66]. Finally, SNPs with MAF over 0.05 and less than 20% missing data were used to compare their ability to assess population structure (Q) and relative kinship (K) for the Chinese and U.S. association panels separately.

Structure 2.3.4 was used to estimate population structure and to assign genotypes to subpopulations using SNP data [67]. The membership coefficients for each individual in each subpopulation for the two association panels were calculated by an admixture model with a burn-in length and MCMC (Markov chain Monte Carlo) of 10,000 [67, 68], with the number of subpopulations (K) ranging from 1 to 15 and for each K in the pilot test with 15 iterations. A K value was determined by finding a sharp increase for the mean value of LnP(D) against K. Then a detailed run (K = 1–6) was conducted with the same model a burn-in length of 50,000 followed by 50,000 iterations. 20 repeats were set to quantify the variation of the likelihood. K was selected as the highest value of ΔK according to the formula proposed by Evanno et al.:

ΔK=m(|L(K+1)−2L(K)+L(K−1)/s[L(K)]

where m is the mean value of K and s means standard deviation for K over runs [37]. Furthermore, the PCA analyses of the 188 lines were conducted by using GAPIT based on the SNP genotyping results [69].

The relative kinship was calculated by SNP markers conducted within software package SPAGeDi version 1.0 [70]. All negative values between individuals were set to 0, indicating that they are not related [29]. Marker profiles by the Bayesian clustering method of STRUCTURE 2.3.4 were used to analyze genetic relationships within association panels.

### Candidate genes based genotyping

Seven SSR markers, R1-2, R7B-2, B-4, STS-8, STS-11, STS-12, and Trx-1 were used for genotyping the *Scmv1* region in both the Chinese and the U.S. panel [25]. For detection of SSR and STS markers, PCR reactions were prepared in a 10 μL reaction volume containing 100 ng of genomic DNA, 1 μL of 10 × PCR buffer, 0.2 mM of each dNTP, 0.2 μM of each forward and reverse primer, and 1 U of Taq polymerase (Thermo Scientific, Waltham, MA, USA). PCR reactions were performed using a PTC-200 Peltier Thermal Cycler (MJ Research, St. Bruno, Quebec, Canada) with particular annealing temperature and elongation duration adapted to each reaction. The PCR products were subjected to electrophoresis using 6% polyacrylamide gels, amplification products were visualized by silver staining.

12 SSR markers were used for *Scmv2* genotyping of the U.S. panel, designed by PRIMER3 (http://gmdd.shgmo.org/primer3/?seqid=47) based on B73 sequence and previous work on *Scmv2* fine mapping (Table 4), while 5 out of these 12 SSRs were used for genotyping the Chinese panel. SSRs were amplified via PCR with fluorescently labeled primers in 20 μL reactions containing 40 ng genomic DNA, 2 μL of 10 × PCR buffer, 1.5 mM MgCl_2_, 0.2 mM dNTP, 0.2 μM labeled forward and unlabeled reverse primer, and 0.5 units of Taq polymerase (Thermo scientific, Waltham, MA, USA). The lengths of PCR products were obtained by electrophoresis on an Applied Biosystems 3730 DNA Analyzer (Applied Biosystems, Carlsbad, CA, USA) at the DNA Facility of Iowa State University (http://www.dna.iastate.edu/). Software package Peak Scanner 1.0 (Applied Biosystems, Carlsbad, CA, USA) was used to record fragment sizes, which were re-checked manually.

**Table 4:** SSR markers used for association analysis in the *Scmv2* region.

Primer	Forward (5'-3')	Reverse (5'-3')
**184B1**	GAGCACAAAACCGAAGGGTA	CCGAAGGTGATTAGAGGGCTA
**DJF004**	ATATCCGGATCCATCCAGTG	TTGTGTTGCTGTTGCGTACA
**184FG018**	GACTCAACTCAAGGATGG	TGTTCAGCCAGAAGAGAG
**212FG008**	GGTGGAGGAGGTAACTCAAGAC	GCAGTGCAGAAGGAAACCAT
**DJF003**	AGGCAATCCTGCTCGAATAA	AGCCTAGGGCTAGCAAGGTC
**197-S06**	ACCGAAGTTGACATGGGAAG	CAGGAAGCAAGGCAGTTGAT
**202S05**	GCGCGGTACTTCTCAATCTC	GGCTACGACGAAAACCAGAA
**202A1short**	GCGTAGCCTAGCACATTATG	CCACATAGACCTAGCAGCAA
**2098–5**	CGAGGAAGCAGATGAAGGAG	GCAGTGCAGAAGGAAACCAT
**207FG003**	CGATCCACACCAGGTAAAGG	CAATTTCCTACCCACCGAAA
**Bnlg1601**	CAGACCAGAGACCATCTGCA	ATCGTGCGCTAGTCCAGAGT
**Auxin**	CGTTGATAAGAGAGGAGAGC	ACTTAGCAGTGCTGGTCTCA
**184-GS17**	TTGTGTTGCTGTTGCGTACA	ATATCCGGATCCATCCAGTG

### Association mapping

The TASSEL 3.0 software package (http://www.maizegenetics.net/bioinformatics/tasselindex.htm) was used to identify SSRs in the *Scmv1* and *Scmv2* regions that were associated with SCMV resistance. Associations of the 3072 SNPs with SCMV resistance were also tested in this study. For both regional and genome-wide associations, a correction for multiple testing was performed by using Bonferroni’s procedure; *P* = 2 ×10^−6^ for genome-wide association of SNPs, *P* = 0.007 for the *Scmv1* and *P* = 0.004 for *Scmv2* region, respectively. These *P* values were considered to be indicative of significant marker associations with SCMV resistance. To control for both Type I and Type II errors, a mixed linear model (MLM) incorporating population structure (Q) and pairwise kinship matrix (K) were performed using TASSEL 3.0 [29, 71]. The two association panels were analysed separately because of the different phenotypic data collection methods. However, a joint analysis of the two panels associated with SCMV-BJ resistance at 1 WPI was also conducted. As epistasic interactions between *Scmv1* and *Scmv2* have been reported [16, 23], we intended to test for digenic epistasic interactions of allele combinations for markers in the *Scmv1* and *Scmv2* regions. However, only multiallelic SSR markers were available in those regions, with six alleles for Trx and seven alleles for STS-8 in the *Scmv1* region, six alleles for DJF004 and five alleles for 207FG003 in the *Scmv2* region. As allele classes for these markers in the *Scmv1* and *Scmv2* regions contained too few individuals, we did not have sufficient statistical power to detect epistasis. For this reason, we decided to not test for presence of epistasis in this study.

### Statistical analysis

Analysis of variance (ANOVA) was conducted by SAS Version 9.1 (SAS Institute) for the preliminary statistical analysis of trait data using a GLM procedure, separately for panels, isolates, and time points. The mixed procedure for analysis of variance by SAS was:

$$Y_{ijl}=\mu +E_{i}+L_{j}+EL_{ij}+\varepsilon_{\left(ij\right)l}$$

Where: *Y*_*ijl*_ represents the observation from the *ijl* ^*th*^ plot, *μ* is the overall mean. *E*_*i*_ is the experiment; *L*_*j*_ is the lines; *EL*_*ij*_ is the interaction; ε means error. Lines were considered to be fixed effects, experiment was considered to be random as well as *ε*_*(ij)l*_ and the interaction. This ANOVA model was applied to each time point. Genotypic (*V*_*G*_), and phenotypic (*V*_*P*_) variance as well as broad sense heritability (*H* ^2^) were all calculated on mean basis. Heritability was calculated as follows:

$$H^{2}=\frac{V_{G}}{V_{P}},V_{G}=\frac{MS_{G}-MS_{E}}{rep},V_{P}=\left(\frac{MS_{G}-MS_{E}}{rep}\right)+MS_{E},H^{2}=\frac{\frac{MS_{G}-MS_{E}}{rep}}{(\frac{MS_{G}-MS_{E}}{rep})+MS_{E}}$$

MS_G_ and MS_E_ stand for mean square of genotype and mean square error, respectively. Rep is the number of independent replications (2). Adjusted means and effective error mean squares from each experiment were used to compute combined analyses of variances across experiments. TASSEL 3.0 was used to perform a Mixed Linear Model (MLM) analysis, incorporating trait, population structure (Q), and kinship matrix (K) data [71].

## Supporting Information

S1 Fig The distribution histogram of the U.S. panel phenotypic data challenged with different SCMV isolates and experiments. A: SCMV-Seehausen at Experiment 1; B: SCMV-Seehausen at Experiment 2; C: SCMV-BJ at Experiment 1; D: SCMV-BJ at Experiment 2.
(TIF)

S2 Fig Plot of the first two principal components (PC1 and PC2) of the 188 lines. Green: the Chinese panel; Red: the U.S. panel.
(TIF)

S1 Table Members in each sub-groups for both U.S. and Chinese panel defined by Structure. (DOCX)

S2 Table Information for the maize lines in the U.S. association panel panel, including the source of these lines. (DOCX)

S3 Table 3072 SNP markers information and genotyping results. (XLSX)

## Abbreviations

SCMV: sugarcane mosaic virus
SNP: single nucleotide polymorphism
SS: Stiff Stalk Synthetic
NSS: Non Stiff Stalk Synthetic
SSR: single sequence repeat
STS: sequence-tagged site
MDMV: maize dwarf mosaic virus
PCR: Polymerase chain reaction
Q: Population structure
GLM: General linear model
MLM: Mixed Linear Model
