# Supplementary figures and images for: Characterization of Sugarcane Mosaic Virus Scmv1 and Scmv2 Resistance Regions by Regional Association Analysis in Maize
Source: PLoS One. 2015 Oct 21;10(10):e0140617. doi: 10.1371/journal.pone.0140617 (PMC4619251; doi:10.1371/journal.pone.0140617)

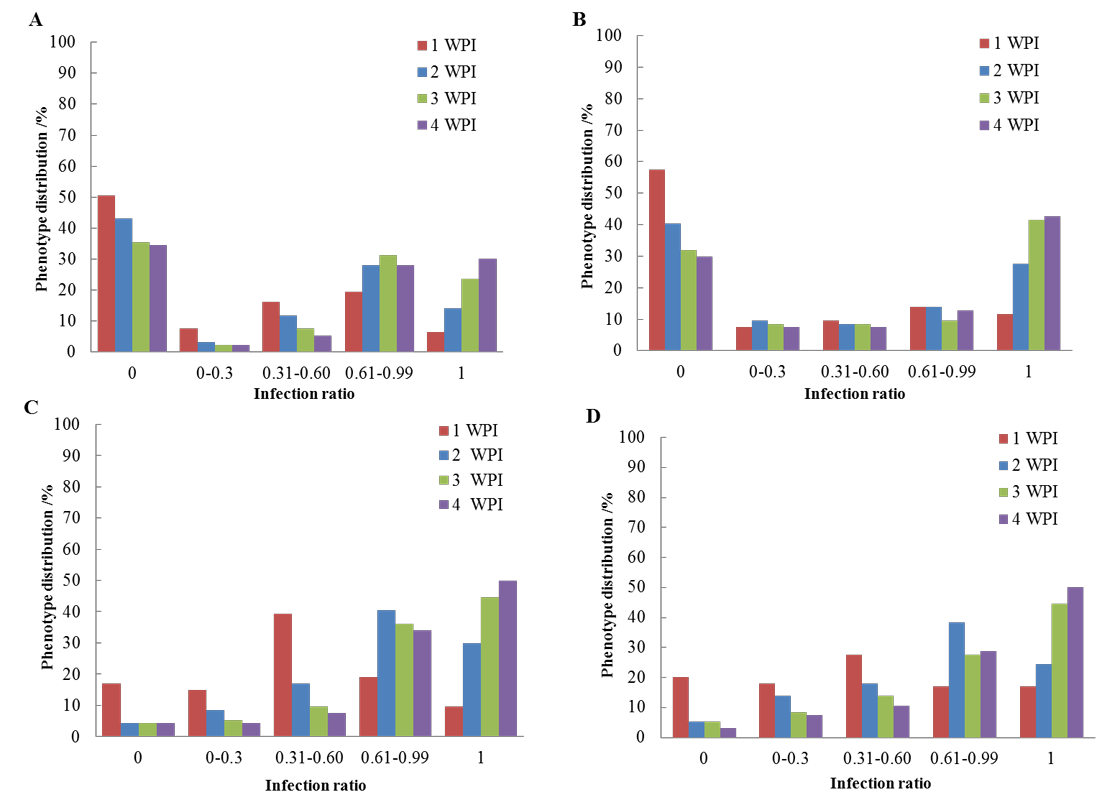

Supplement: S1 Fig — A: SCMV-Seehausen at Experiment 1; B: SCMV-Seehausen at Experiment 2; C: SCMV-BJ at Experiment 1; D: SCMV-BJ at Experiment 2. (TIF) [file pone.0140617.s001.tif]

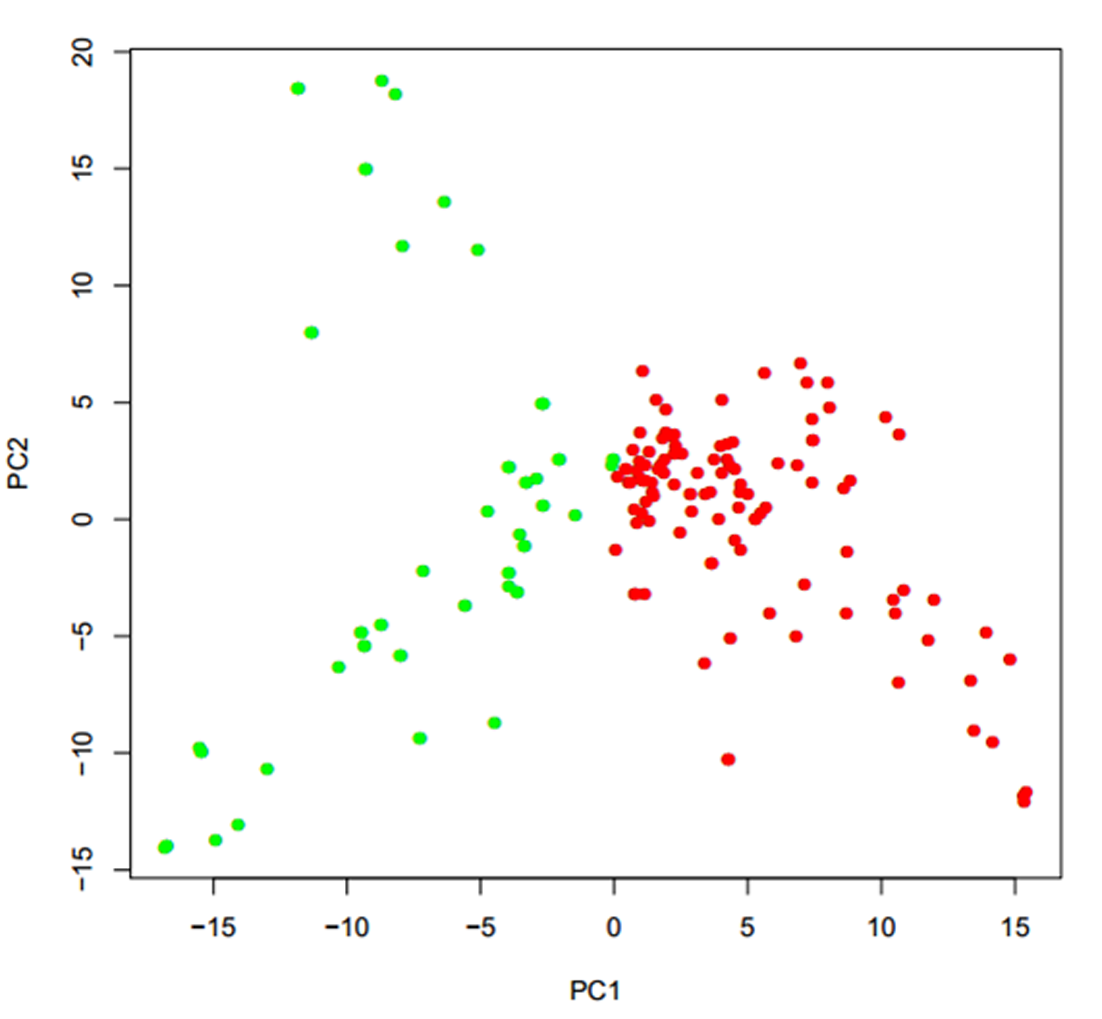

Supplement: S2 Fig — Green: the Chinese panel; Red: the U.S. panel. (TIF) [file pone.0140617.s002.tif]
