# Supplementary material for: Characterization of Sugarcane Mosaic Virus Scmv1 and Scmv2 Resistance Regions by Regional Association Analysis in Maize
Source: PLoS One. 2015 Oct 21;10(10):e0140617. doi: 10.1371/journal.pone.0140617 (PMC4619251; doi:10.1371/journal.pone.0140617)
Supplement: S1 Table — (DOCX) [file pone.0140617.s003.docx]

| **Sub-groups** | | **Lines** |
| --- | --- | --- |
| **U.S. panel** | **P1** | A632, A634, B14, B37, B47, B68, B73, B84, CM105, D145, FAP1360A, FAP1396A, FAP954A, I114H, KS22, LH1, LH119, LH132, LH74, PB80, PHG35, PHG39, PHG86, PHW52, PHK29, R2306 |
|  | **P2** | 10940, A188, AB28A, B97, CML103, CML228, CML247, CML277, CML322, CML333, CML52, CML69, Co125, Co158, D06, D09, D21, D32, EP-1, F7, H99, HBA1, Hix4243, Hix4283, HP301, ICAL210, ICAL224, Ki11, Ki3, Ky21, LH38, LH51, LH59, LH82, LH85, M162W, M37W, Mo17, Mo18W, Mo22, MP705, MS71, NC350, NC358, Oh1V1, Oh28, OH43, OH7B, P39, P737, Pa405, PH207, PHG29, PHG47, PHG50, PHG84, PHJ40, PHK05, PHK76, PHR25, PHV78, PHZ51, Sh2, T115, Tx303, Tzi8, Va85, W117 |
| **Chinese panel** | **P3** | 10940, 1121, 137, 478, 5237, 4F1, 5003, 515, 52106, 5311, 6407, 698-3, 7286, 7296, 7364, 7397, 7416, 7922, 8001, 8701, 9508, A188, Ay3566, Ay420, B73, BT1, Chang3, Chang72, Dan598, Dan9046, Danhuang02, Dong46, F7, FAP1360A, FEB-48, H99, Huotanghuang, Ji 69, Ji 846, Ji 853, Ji4112, Ji63, Ji842, K10, K12, K22, Longkang11, Lv28, Mo17, Nan213, Q1261, Qi205, Shen137, Siyi, Va35, Wu109, Yan414, Ye107, Ye832, Yu374, Zheng22, Zheng32, Zheng58, Zi330, Zong31 |
|  | **P4** | 1145, 122, 178, 444, 6256, 7314, 7368, 77, 7884, 8112, A50, Chang7-2, Dan340, Dan599, Hai9-21, HuangC, Huangyesi, Huangzao4, Ji53, Jiao3, Jiao51, Jun971, Lian87, LX9801, P138, Q1537, Qi319, Xi502, Zi341 |
