# Supplementary material for: Characterization of Sugarcane Mosaic Virus Scmv1 and Scmv2 Resistance Regions by Regional Association Analysis in Maize
Source: PLoS One. 2015 Oct 21;10(10):e0140617. doi: 10.1371/journal.pone.0140617 (PMC4619251; doi:10.1371/journal.pone.0140617)
Supplement: S2 Table — (DOCX) [file pone.0140617.s004.docx]

| **Line** | **Origin** | **Line** | **Origin** | **Line** | **Origin** |
| --- | --- | --- | --- | --- | --- |
| FAP1360 | Europe | LH59 | US, Iowa | Ki3 | Thailand |
| F7 | Europe | LH85 | US, Iowa | Ky21 | US, Kentucky |
| Pa405 | US, Pennsylvania | LH119 | US, Iowa | T115 | US, Tennessee |
| 10940 | Europe | PHG29 | US | Tx303 | US, Texas |
| D32 | Europe | PHG35 | US | Tzi8 | Nigeria |
| D21 | Europe | PHG84 | US | OH7B | US, Ohio |
| B68 | US , Iowa | PHG86 | US | Oh43 | US, Ohio |
| FAP954A | Europe | PHJ40 | US | Ab28A | US, Alabama |
| H99 | US, Indiana | PHZ51 | US | A632 | US, Minnesota |
| D09 | Europe | PHW52 | US | A634 | US, Minnesota |
| FAP1396 | Europe | PHK29 | US | M37W | South Africa |
| D06 | Europe | PHR25 | US | Ms71 | US, Michigan |
| R2306 | unknown | PHK76 | US | NC350 | US, Iowa |
| D145 | Europe | PHG50 | US | NC358 | US, North Carolina |
| Co125 | Europe | PHG39 | US | P737M20 | US, Michigan |
| Co158 | Europe | PHG47 | US | Va85 | US, Virginia |
| EP-1 | unknown | PHK05 | US | I114H | US, Illinois |
| A188 | US, Minnesota | PHV78 | US, Illinois | P39 | US, Indiana |
| B73 | US, Iowa | CML103 | Mexico | Hp301 | US, Indiana |
| Mo17 | US, Missouri | CML228 | Zimbabwe | Oh1V1 | US, Ohio |
| B37 | US, Iowa | CML247 | Mexico | Oh28 | US, Ohio |
| B14 | US, Iowa | CML277 | Mexico | ICAL210 | unknown |
| B84 | US, Iowa | CML322 | Mexico | PH207 | US, North Dakota |
| B47 | US, Iowa | CML333 | Mexico | KS22 | Korea |
| B97 | US, Iowa | CML52 | Mexico | HiX4283 | US, Hawaii |
| LH1 | US, Iowa | CML69 | Mexico | Hix4243 | US, Hawaii |
| LH51 | US, Iowa | CM105 | Mexico | Mp705 | US, Mississippi |
| LH82 | US, Iowa | M162W | South Africa | ICAL224 | unknown |
| LH132 | US, Iowa | Mo18W | US, Missouri | W117 | US, Wisconsin |
| LH74 | US, Iowa | Mo22 | US, Missouri | Sh2 | US, Wisconsin |
| LH38 | US, Iowa | Ki11 | Thailand | HBA1 | US, Illinois |
|  |  |  |  | PB80 | US |
